# A Dyadic Behavioral Intervention to Optimize Same Sex Male Couples’ Engagement Across the HIV Care Continuum: Development of and Protocol for an Innovative Couples-based Approach (Partner Steps)

**DOI:** 10.2196/resprot.6271

**Published:** 2016-08-25

**Authors:** Angela Robertson Bazzi, Kirkpatrick B Fergus, Rob Stephenson, Catherine A Finneran, Julia Coffey-Esquivel, Marco A Hidalgo, Sam Hoehnle, Patrick S Sullivan, Robert Garofalo, Matthew J Mimiaga

**Affiliations:** ^1^ Boston University School of Public Health Department of Community Health Sciences Boston University Boston, MA United States; ^2^ School of Medicine University of California, San Francisco San Francisco, CA United States; ^3^ School of Nursing Department of Health Behavior and Biological Sciences University of Michigan Ann Arbor, MI United States; ^4^ The Center for Sexuality and Health Disparities University of Michigan Ann Arbor, MI United States; ^5^ School of Medicine Emory University Atlanta, GA United States; ^6^ The Fenway Institute Fenway Health Boston, MA United States; ^7^ Feinberg School of Medicine Department of Pediatrics Northwestern University Chicago, IL United States; ^8^ Feinberg School of Medicine Department of Psychiatry and Behavioral Sciences Northwestern University Chicago, IL United States; ^9^ Ann & Robert H. Lurie Children’s Hospital of Chicago Division of Adolescent Medicine Chicago, IL United States; ^10^ Rollins School of Public Health Department of Epidemiology Emory University Atlanta, GA United States; ^11^ Feinberg School of Medicine Department of Preventive Medicine Northwestern University Chicago, IL United States; ^12^ School of Public Health Department of Behavioral and Social Health Sciences Brown University Providence, RI United States; ^13^ School of Public Health Department of Epidemiology Brown University Providence, RI United States; ^14^ Albert Medical School Department of Psychiatry and Human Behavior Brown University Providence, RI United States; ^15^ Institute for Community Health Promotion Brown University Providence, RI United States

**Keywords:** HIV prevention, interventions, men who have sex with men, HIV cascade, HIV care continuum, ART adherence, engagement in care, couples-based interventions, intervention development

## Abstract

**Background:**

An estimated one- to two-thirds of new human immunodeficiency virus (HIV) infections among US men who have sex with men (MSM) occur within the context of primary partnerships. Thus, HIV interventions that recognize and harness the power of relationships are needed. Increasingly, HIV prevention efforts are being directed toward improving engagement across the HIV care continuum from testing to linkage to care, antiretroviral therapy (ART) adherence, engagement in care, and viral suppression. However, to our knowledge, no behavioral interventions have attempted to address the HIV care continuum using a dyadic approach.

**Objective:**

The objective of this paper is to describe the development of and protocol for an innovative couples-based approach to improving treatment adherence and engagement in care among HIV serodiscordant and concordant HIV-positive same sex male couples in the United States.

**Methods:**

We developed the Partner Steps intervention by drawing from relationship-oriented theory, existing efficacious individual-level ART adherence interventions, couple-focused HIV prevention interventions, and expert consultation. We incorporated new content to address all aspects of the HIV care continuum (eg, linkage to and retention in care) and to draw on relationship strengths through interactive activities.

**Results:**

The resulting theory-based Partner Steps intervention is delivered by a trained bachelors-level counselor (interventionist) over 2 in-person sessions with male-male dyads in which at least 1 partner has recent suboptimal engagement in HIV care. Each session is designed to use relationship strengths to increase motivation for HIV care and treatment, and cover sequential intervention “steps” relating to specific challenges in HIV care engagement and barriers to ART adherence. For each step, couples work with a trained interventionist to identify their unique challenges, actively problem-solve with the interventionist, and articulate and commit to working together to implement a plan in which each partner agrees to complete specific tasks.

**Conclusions:**

We drew on theory and evidence to develop novel intervention strategies that leverage strengths of relationships to address engagement across the entire HIV care continuum. We provide details on intervention development and content that may be of use to researchers as well as medical and mental health professionals for whom a dyadic approach to HIV prevention and care may best suit their patient population.

## Introduction

Men who have sex with men (MSM) experience the highest risk for human immunodeficiency virus (HIV) acquisition in the United States [1], accounting for 64% of new infections in 2012 [2], up from 53% in 2006 [3]. Substantial evidence supports the role of primary partnerships in increasing HIV transmission risk among MSM, with an estimated one- to two-thirds of incident HIV infections among MSM attributed to sexual behavior with main partners [1,4-9]. Increased HIV risk within primary partnerships has been attributed to greater frequency of unprotected anal sex with main versus casual partners, lowered perceived risk within relationships and reduced HIV testing [10-18], and desires to demonstrate intimacy, trust, and commitment [7,8,19-23]. These relationship dynamics are associated with lower self-perceived HIV risk and suboptimal HIV testing among MSM in committed relationships [24,25], potentiating the prevalence of undiagnosed infections among male-male couples [26]. In addition to reducing morbidity and mortality from HIV infection, diagnosing HIV and linking HIV-infected individuals to care and initiating antiretroviral therapy (ART) greatly reduces the risk of onward transmission to their partners [27,28]. Despite the increasing interest in a couples-focused approach to HIV prevention among MSM [29-32], to date, no theory-based behavioral interventions exist to promote male-male couples’ engagement in the HIV care continuum from linkage to retention in HIV care. This paper describes the development of a novel theory- and evidence-based intervention, Partner Steps, designed to promote and sustain engagement across the HIV care continuum among HIV serodiscordant and concordant HIV-positive male-male couples.

The HIV care continuum is a model for describing progression from HIV diagnosis to the achievement of controlled viremia in the body known as viral suppression [33,34]. Four stages are generally recognized in this continuum, including (1) diagnosis with HIV infection, (2) engagement in HIV medical care, (3) prescription of ART medications, and (4) achieving viral suppression (viral load <200 copies/mL) [35-37]. Individuals progress through these stages at different rates and may experience multiple challenges along the way (eg, transportation, communicating with providers, maintaining a Daily Medication Schedule; see Table 1 for a comprehensive list). The significance of these challenges is apparent in the observed percentage drops at each successive stage of this continuum. In 2011, an estimated 86% of all HIV-infected individuals in the United States had been tested and were aware of their HIV status [35,37]. Among those diagnosed with HIV, approximately 40% were engaged in HIV medical care. Of those engaged in HIV care, only 37% had been prescribed ART [35,37]. Although detailed national data on ART adherence are not available, studies in a variety of populations have documented a wide range of adherence (53-89%) [38-48], with an estimated national average of 70% [49]. With high ART adherence recommended for achieving viral suppression, the suboptimal levels observed may help explain why viral suppression, the ultimate end point of the HIV care continuum, is believed to be achieved by only 30% of the HIV-infected population [35,37]. It is also important to note that while the continuum is presented as a linear stage progression, it in fact represents a series of dynamic states. For example, some individuals may achieve and then lose viral suppression while others may experience periods in which they are retained and then absent from HIV care.

With viral suppression improving the health of HIV-infected individuals and reducing the likelihood of further transmission to partners, improving outcomes along the entire HIV care continuum has been identified as an important HIV prevention priority [50]. Recommendations from the US Preventive Services Task Force include screening all patients aged 15 to 65 for HIV. Moreover, in light of conclusive evidence that viral load suppression via ART helps prevent onward transmission to sex partners, the US Department of Health and Human Services guidelines now recommend prescribing ART medications to every adolescent and adult diagnosed with HIV regardless of disease progression status [50]. This shift away from the previous use of CD4 levels as a criteria for starting ART–in conjunction with expanded access to health care through the Affordable Care Act–has changed the climate in HIV prevention [51-53]. Increasing efforts are now being directed toward the development of innovative approaches to improving engagement along the HIV care continuum, particularly for populations most affected by HIV, including MSM, transgender women who have sex with men, and racial/ethnic minorities [35,50]. As main partner transmission accounts for an estimated one- to two-thirds of incident HIV infections among MSM, main-partner dyads could benefit greatly from an intervention centered on improving their engagement across the HIV care continuum [1,4-9].

Most prior interventions with male couples focused on the first stage of the HIV care continuum, getting couples tested, have been largely successful. For example, couples HIV testing and counseling (CHTC) is a particularly promising intervention for the earliest stage of the continuum, HIV diagnoses through testing. CHTC has been used as an HIV prevention intervention in Africa and other regions for over 20 years [54] and is considered by the US Centers for Disease Control and Prevention (CDC) to be a “high leverage HIV prevention intervention” [55]. The critical difference between the CHTC model and the conventional model of individual HIV testing and counseling is that partners receive HIV testing together and combined counseling and prevention messaging based on the characteristics of their relationships and their joint HIV status. CHTC has been adapted for US MSM and has been found to be highly acceptable [54,56]. Qualitative data from MSM in 3 US cities shows strong support among MSM for CHTC, including its use in forming sexual agreements (ie, rules regarding outside sexual partners) and communicating about sexual risk behaviors and condom use [57]. Recent work has both shown CHTC is highly acceptable to male-male couples [58] and demonstrated CHTC’s safety, with safety measured as no increases in relationship dissolution or intimate partner violence among male couples undergoing CHTC [59]. CHTC is now endorsed by the CDC, and has been rolled out in over 40 US states [60]. Until now, few interventions have delved further into the HIV care continuum (eg, engagement in care). Instead, most of the limited number of dyadic interventions emphasize a single stage in the HIV care continuum or are not tailored to the specific needs of male-male couples. In particular, the handful of intervention studies that have worked with couples to improve ART adherence [61] were not specifically designed for male-male couples and did not address important issues upstream in the HIV care continuum [31,61,62].

Partner Steps is a counseling intervention designed to address multiple stages of the HIV care continuum within the male-male dyad [32,62]. Recent findings indicate that such an intervention would be acceptable among same-sex male couples. Findings from focus groups conducted by Goldenberg et al [32] suggest male-male couples may be interested in more comprehensive care at the level of the dyad. Many couples indicated that comprehensive dyadic care had the potential to strengthen positive relationship dynamics [32]. In addition, there is evidence that dyadic interventions result in better ART adherence when compared with individual adherence counseling interventions. For example, in a randomized controlled trial of 215 couples, including male-male couples, HIV-infected persons receiving ART adherence counseling with their partners had significantly higher levels of ART adherence compared with those who did not [61]. Similarly, among a cohort of 210 male-male HIV-serodiscordant couples, both the patient’s positive appraisal of his relationship and his partner’s positive beliefs regarding treatment self-efficacy were linked to greater self-reported ART adherence; although, in this study, participants did not receive counseling as a dyad [63]. Working with couples offers unique advantages for designing an intervention: couples can cope together to reduce stress burden; work in tandem to remember routine responsibilities; problem solve more effectively; pool resources; and navigate social environments as a unit. These advantages in addition to reported interest in couples-based interventions serve as the impetus behind Partner Steps.

With no interventions to date attempting to address every stage of the continuum for broadly defined male-male couples (in which at least one partner is HIV positive) and evidence that such an intervention would be acceptable among male-male couples [31,32,62], we developed Partner Steps for HIV serodiscordant and concordant HIV-positive male-male couples by adapting selected content from efficacious adherence interventions at the individual level. We also developed entirely new content and approaches for working with male dyads. The aim of this article is to describe our intervention development process in order to inform other investigators and provide recommendations for programs in HIV prevention and treatment.

## Methods

### Overview and Specific Aims

Partner Steps is designed to increase couples’ motivations, behavioral and problem-solving skills, mutual support, and collaboration with the ultimate goal of promoting engagement in HIV care and ART adherence. We developed this theory-based intervention with attention toward leveraging relationship strengths within a diverse community of HIV serodiscordant and concordant HIV-positive male-male couples at any stage of engagement in HIV care, including those who are HIV care–naive. The intervention development process involved drawing from both theoretical and evidence bases to consider men’s relationship dynamics throughout all components of the resulting Partner Steps program. All of the research described in this multisite project received ethics approval from the institutional review boards of Emory University in Atlanta, GA, Fenway Health in Boston, MA, and the Ann & Robert H. Lurie Children’s Hospital in Chicago, IL.

### Theoretical and Evidence Base

Several promising models for addressing particular components of the HIV care continuum have been developed and tested in other contexts and populations. To identify and develop an intervention relevant to improving couples’ HIV care outcomes, we critically assessed prior ART adherence interventions for HIV-infected individuals including Safren and colleagues’ [64] influential Life-Steps intervention. Over the past decade, Life-Steps has been adapted for diverse settings and populations leading to content and structure that differs with respect to target behaviors, dose (eg, length, number of sessions), interventionist background and training, and assessment and follow-up techniques [65,66]. Notably, members of our study team recently adapted Life-Steps for HIV-infected adolescents, which required greater emphasis on the role of family, peer, and other interpersonal relationships and social contexts [65]. Building on this work we developed content and structure appropriate for HIV serodiscordant and concordant HIV-positive male-male couples.

Life-Steps integrates general principles of cognitive behavioral therapy, motivational interviewing, and problem-solving [67] to help patients solve “blocks” of adherence problems through a step-by-step approach [64]. To the extent possible and relevant, we preserved the overall structure of this efficacious intervention, in which a trained interventionist engages participants in a series of educational, problem-solving, and rehearsal strategies to identify specific barriers to ART adherence and develop solutions through a series of small, manageable problem-solving “steps.” This process was designed to train individual patients to identify and address complex and stressful challenges of medication adherence into simplified, proximal steps addressed in a checklist format [64]. For the unique social and relationship contexts that characterize male-male couples’ experiences, we adapted, reorganized, and expanded the original 10 steps to involve both partners in identifying barriers to and developing solutions for improved ART adherence and engagement in HIV care.

We subsequently reviewed the existing literature’s evidence from observational and intervention studies focusing on medication adherence and engagement in HIV care separately and for individuals. Absent from these programs was a focus on social and relationship dynamics; we thus drew from multilevel frameworks [68,69] and theories of interpersonal interaction to help conceptualize adaptions that would be necessary given the unique social experiences and relationship dynamics of male-male couples. Lewis et al’s [69] conceptual model of couple’s health behavior change incorporates theories of communal coping and interdependence to understand how relational and dyadic processes determine behavior change, particularly in the uptake of risk-reducing behaviors. This model posits that the couple’s interdependence influences behavior as motivations shift from what is best for each partner’s wellbeing to what is best for the relationship. Using this construct, the adaptations or additional steps centered on building communal social skills by harnessing a couple’s motivation to keep their relationship healthy, thereby increasing the likelihood of adopting positive health behavior change and sustaining this change as they work together. When adapting and enhancing the original 10 steps to a dyadic context, we drew from these two perspectives in order to infuse Partner Steps with adequate consideration of how interpersonal interactions could be used to increase couples’ motivation for engagement in HIV care. We also reviewed the counseling principles that inform existing couples-based sexual health and primary HIV prevention interventions [70-72], including CHTC [50,54,56]. This relationship-oriented theoretical framework guided our review and adaptation of existing individual-based intervention components.

### Adaptation of Existing Evidence-Based Interventions for Couples

We adapted existing steps for a context in which a trained interventionist meets with both partners jointly, equally engaging both partners in the session despite the desired outcome of the curriculum being optimal ART adherence for the HIV-infected partner. For example, the first original step of Life-Steps provided information to individual patients on the importance of ART adherence for improved individual health outcomes (eg, through reduced viral load) via a video followed by discussion with the interventionist [73]. To increase the relevance of this background information and motivation for HIV-negative partners to help their HIV-positive partners with adherence, we updated and revised the content of this step to emphasize how optimal adherence and engagement in care can reduce transmission, simultaneously protecting the HIV-negative partner from transmission and improving the health of the HIV-positive partner [27,28]. Rather than employ the original video format, we elected to deliver the information through an engaging conversation, which required each partner to interact with the interventionist and with one another (eg, after delivering a key message, the interventionist asks one partner to explain, in his own words, to the other partner).

While increasing dyadic interaction and collaboration, these adaptations to leverage interpersonal dynamics and strengths are still in line with Life-Steps’ utilization of effective approaches that increase motivation for behavior change [64] and encourage participants to take an active role in developing, practicing, and planning to implement their own solutions to specific adherence problems [73]. One particularly useful method from Life-Steps that we preserved throughout our adaptation is based on problem solving methods [67]. For example, for each adherence barrier, individuals are encouraged to articulate their goal, identify potential barriers to reaching the goal, and develop a plan to overcome the barriers [64]. We preserved the structure of this method but reframed activities and language such that interventionists encourage both partners to work together to articulate a particular goal, identify barriers to reaching that goal, and develop plans to overcome those barriers that recognizes potential stumbling blocks and includes “backup” plans [64]. For example, for the original Life-Steps topic of communicating with providers, we encourage both partners to identify barriers and develop and practice communication strategies together. Partners are also encouraged to attend health care visits together, if possible and appropriate (discussions can be tailored to couples’ unique relationship dynamics, which are explored by the interventionist in the introductory “getting to know your relationship” module).

### Reorganizing Existing Intervention Content to Enhance Focus on Relationship Contexts

We reorganized some of the original Life-Steps content given that we would need to save time to cover additional (new) content covering relationship and social topics, mental health and substance abuse, and linkage to and engagement in HIV care, as described below (see Multimedia Appendix 1). Due to the increased amount of content, we planned a two-session intervention instead of the original single-session format of Life-Steps. For the first “adherence steps” session, we grouped six steps that related to logistical considerations, including getting to appointments (transportation), obtaining medications, communicating with providers, storing and transporting medications, coping with side effects, and developing a concrete daily medication schedule. The overall objective for each of these steps is to enable couples to jointly identify related adherence barriers and, if barriers exist, develop solutions and make specific plans for overcoming the logistical barriers together. For each specific step, we also adapted content and interventionist scripts to better engage HIV-negative partners and facilitate collaboration within couples in the problem solving process.

Based on the literature on male couples and expertise within our team [32,74], we recognized a need to expand upon the original Life-Steps content in order to better address the social contexts and relationship dynamics experienced by male-male couples and created several new, more social-oriented steps. We grouped these four new relationship and social steps into a second “adherence steps” session covering self-care and relationships, communicating within relationships, managing social lives and other interpersonal relationships, and dealing with privacy and disclosure. The objectives for these steps vary from the objectives of the previous set of more logistical steps to focus on and require more nuanced discussion of barriers within these interpersonal domains [32,69,74,75]. Similar to the first session, if couples jointly identify adherence barriers relating to any of these steps (eg, having a social network unaware of the couples’ serodiscordant status), the interventionist then works with the couple to develop solutions and make specific plans that partners can implement together. These modules explore social and relationship influences on adherence and provide partners with a safe space to problem-solve and develop plans for achieving progress toward the ultimate goal of viral load suppression. One exception is the topic of disclosure, for which the interventionist emphasizes respect for individual preferences and works with couples to ensure that they leave with a “shared vision” even if that requires a compromise between partners.

### Expansion of Existing Intervention Content to Promote Engagement in HIV Care

In addition to logistical and social/relationship-focused adherence “steps” described above, we developed new content to address other stages of the HIV care continuum, recognizing that some couples in this population require additional motivation and problem-solving around linkage to care and retention in care over time (see Multimedia Appendix 2). While initial linkage to care (including connecting with a provider for the first time) and sustained engagement in care over time represent two different components of the HIV care continuum, the underlying motivation, potential barriers, and skills necessary to achieve linkage and engagement overlap significantly. We thus combined these two components of the HIV care continuum into the resulting “preadherence steps” session through which partners are counseled together. The overall format of this session (which can stretch over two sessions as necessary) involves new content focused on increasing motivation (ie, background information), problem-solving around a set of identified barriers (ie, steps), and developing a long-term plan for improved engagement in HIV care.

To develop this new content, we first reviewed the literature on HIV care engagement as well as previous quality improvement projects to identify potential barriers and existing efficacious methods for facilitating engagement in HIV care [33,35-37,76-87]. We adapted these previous interventions and recommendations to fit the foundational “steps” structure and the social and relationship context of male-male couples. First, to increase motivation for initial linkage to care and engagement in care over time, we reviewed recent literature on seeing a provider regularly and the impact on long term health outcomes [33,86,88,89]. The resulting motivational background information, similar to the “psycho-education” content included in the original Life-Steps [64] and our updated adherence background information (as described above), explain the importance of connecting with providers to receive information about treatment options, monitoring viral load levels, assessing for possible comorbidities and coinfections requiring additional treatment, and ultimately keeping one’s partner and relationship safe [33,86,88,89]. As all couples could benefit from this motivational information, regardless of progression along the HIV care continuum [33], the study team elected to offer this information to all couples as a core element of the intervention.

Next, we reviewed the literature on recommendations to increase engagement in HIV care as well as barriers to achieving this (eg, housing [90-92]) to develop a set of problem-solving steps for engagement in the HIV care continuum [78-81,83,85,87]. In particular, the International Association of Providers of AIDS Care guidelines for improving entry into and retention in care and ART adherence were central to the development process of the “preadherence steps” [85]. Topics identified in the literature for these steps were carefully considered and adapted for relationship contexts, including coping with HIV diagnosis (or serodiscordant status), obtaining health insurance/coverage, navigating the health care system, scheduling and getting to appointments, increasing comfort with providers, anticipating and coping with medication side effects, and increasing interest in HIV care. To be responsive to couples’ unique situations and allow partners sufficient time to work together to generate and plan for implementing solutions to the problems of most relevance to them, this portion of the session begins with an interventionist-administered checklist in which both partners are asked if they endorse particular engagement challenges. Interventionists then initiate problem solving around only the identified challenges, and partners are encouraged to develop solutions and plans together.

### Additional Content and Structure Considerations

In addition to adapting, reorganizing, and expanding upon existing interventions, we developed new content to be covered at various points during Partner Steps to make it more comprehensive and relevant to diverse male-male couples. First, we incorporated brief background information on antiretroviral pre-exposure prophylaxis (PrEP) for HIV prevention. Given the evidence on PrEP for preventing transmission within serodiscordant couples, we incorporated an explanation of PrEP, using simplified language, into the background (motivational) information portion of the adherence and engagement steps sessions. Second, we incorporated an enhanced focus on mental health and substance use within dyadic contexts as acknowledged, but not explicitly addressed, by the original Life-Steps intervention [64]. Extensive research has shown that substance use behaviors are highly interdependent within intimate couples [75,93,94] and are associated with high risk sexual behaviors [95] including breaking sexual agreements among male couples [96]. Third, we adapted and refined framing, content, language, and structure of all Partner Steps modules to make them relevant for concordant HIV-positive couples. The overall aim of the concordant positive intervention is to move both partners through the HIV care continuum toward viral suppression. The intervention leverages relationship strengths, fosters a spirit of team-based care, and encourages each partner to practice self-care while at the same time supporting the needs and preferences of their partner. Similarly, skill-building activities aim to transform both men into engaged patients and supportive partners: each partner cultivates skills for engaging in HIV care and maintaining adherence while also supporting their partner in doing the same thing. The manual also de-emphasizes background information on primary transmission of HIV infection within relationships to instead place greater emphasis on explaining the importance of treatment for the health of both partners and their overall relationship.

Finally, in order to increase cohesion throughout the comprehensive Partner Steps intervention for any couple, regardless of dyadic HIV status or stage along the HIV care continuum, we streamlined the numerous intervention modules using a singular format which used common language, session elements, and styles (eg, for introductions, check-ins, background information, review/planning sessions at the end, and encouragement of interaction throughout all modules). To increase ease of intervention delivery, which could ideally occur through community health centers (where it has been designed by our team), and HIV testing sites, all content was compiled into a single theory-based intervention (one for serodiscordant couples and another for concordant HIV-positive couples). An interactive, 2-day training was developed to ensure that interventionists understood the program rationale and objectives, mastered the methods involved, and had ample opportunity to practice delivering the intervention components and receive feedback and make improvements in counseling styles and techniques. The training was designed for interventionists who could be HIV counselors, community health workers, social workers, or other health care professionals who have been trained in the protocol. Experts on our team and in the broader antiretroviral adherence field carefully reviewed the manual and related training materials.

## Results

### Overview of Partner Steps Content and Delivery

**Figure 1:**
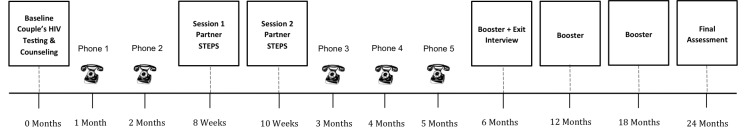
Partner Steps intervention structure and timing.

The resulting theory-based intervention consists of two in-person, manualized intervention sessions delivered to both partners at the same time approximately 2 weeks apart (Figure 1).

Men in the intended target population are ≥18 years of age, biologically male, report being in a “committed” relationship for ≥1 month, and are not experiencing intimate partner violence or coercion to participate. Periodic check-in phone calls are conducted with both partners to assess linkage to care, retention in care, medication uptake and adherence, and relationship dissolution. A trained bachelors-level counselor (interventionist; described below) delivers each intervention session using a Partner Steps manual that structures each session, provides example prose, indicates probing questions and problem-solving approaches, and provides specific activities designed to facilitate active participation from the couple (eg, use of notecards or smartphones; see Table 1). Each session is comprised of a specific topic (eg, background information, communicating with providers) and ends with interactive activities and discussions designed to create a participant-driven plan in which each partner takes ownership and agrees to be responsible for specific tasks (eg, the HIV-negative partner may agree to help with specific activities, as detailed in Table 1).

**Table 1:** Content of the partner steps intervention.

Step	Objectives^a^	Problem Solving	Activities
1. Transportation to appointments	Objective A (if barriers are identified through Objective A, then: Objectives B, C)	Encourage the couple to travel together Encourage the couple to combine medical visits with other errands on the same trip Encourage the couple to ask friends and family for support with transportation Refer the couple to a case manager to assist with transportation discounts or vouchers	Transportation mapping: facilitate an interactive transportation planning session using free, public-access mapping software
2. Obtaining medications	Objective A (if barriers are identified through Objective A, then: Objectives B, C)	Provide couple with medication delivery service options Suggest couple take turns picking up medications Encourage couple to set up auto-refills Encourage couple to set reminders to go to the pharmacy	Facilitated discussion: have each partner turn to each other and work together to come up with a plan to pick up medications regularly
3. Communicating with providers	Objective A (if barriers are identified through Objective A, then: Objectives B, C)	Encourage both partners to attend the visit. Discuss the merits of having 2 people asking questions and 2 people listening to answers Encourage both partners to come up with questions for the provider ahead of time	Writing exercise: have each partner write down questions for the provider on a 3×5 notecard or on their smartphone during the session
4. Storing and transporting medications	Objective A (if barriers are identified through Objective A, then: Objectives B, C)	Encourage the couple to bring a spare dose with them regularly or to store a dose in a strategic location (eg, in a car, work desk, or partner’s house) Suggest the couple plan ahead for long nights out or staying somewhere other than regular residence (traveling) by bringing the appropriate number of doses along	Summarization: solicit opinions on strategies discussed from both partners
5. Coping with side effects	Objective A (if barriers are identified through Objective A, then: Objectives B, C)	Encourage the couple to discuss experiences with side effects with their provider Encourage partners to be open with each other about experiences with side effects Suggest potential routine adjustments (eg, getting more sleep, drinking fluids, exercise, or relaxation practices)	Couples coping brainstorm: facilitate a brainstorming session where couples come up with and reflect upon fun activities they do at home that could be used when side effects are pressing (eg, movie night, game night, staying in)
6. Having a daily medication schedule	Objective A (if barriers are identified through Objective A, then: Objectives B, C)	Suggest other partner take a multivitamin or similar routine to taking medications Suggest the couple combine and integrate pill taking routine with other routines (eg, morning routines, regular schedules) to develop associations between the activities Encourage the couple to use pill boxes Encourage the couple to use alarms	Dot stickers: hand out dot stickers to the couple and explain how they place these in strategic locations around the house to help remind them about taking their medications; Phone reminders: have couple schedule in a recurring reminder on their phones (discretely) to take medications while they are in the session
7. Adherence, self-care, and your relationship	Objectives D, E, F, G, and H	Encourage couple to identify relationship strengths that could be useful for adherence If applicable, promote collective coping mechanisms in place that help alleviate mood considerations If applicable, promote teamwork in reducing the influence of substances on adherence	Reflection: have the couple reflect on self-care concerns (eg, mood, substance use, etc) and relationship-care concerns (eg, collective mood, routines, behaviors, etc) and how these might impact medication adherence
8. Communicating within your relationship	Objectives D, E, F, G, and H	Suggest couples write down discussion topics before discussing with each other Facilitate a discussion about signs and signals that each person feels they are “heard” Facilitate a discussion about each partner’s ideal communication style	Role play: have each partner turn to the other and identify when, where, and how it will be appropriate to discuss adherence. Then have each model an adherence discussion opener.
9. Managing your social life and other relationships	Objectives D, E, F, G, and H	Encourage couple to pool collective knowledge about people in their life to create a plan to cope with social support concerns Create an adherence plan for going out to parties or traveling on weekends Facilitate a discussion about how each partner could balance the various social support systems available to them	(No activity)
10. Dealing with privacy and disclosure	Facilitate discussion of the couples’ privacy and disclosure plan	Facilitate a detailed discussion about who the couple is willing to disclose to or not disclose to	(No activity)
	Facilitate the couples’ ability to problem-solve around privacy and disclosure concerns that serve as barriers to adherence	Provide storage and transportation ideas (eg, secret locations, containers, etc)	
	Create a participant-driven plan and a backup plan to implement in the future to ensure couple leaves session two with a common vision for dealing with privacy and disclosure		

^a^Objectives: A: To identify “step”-relevant barriers to successfully executing “steps” 1-6; B: To facilitate the couples’ ability to overcome “step”-relevant barriers through participant-driven problem-solving; C: To create a participant-driven plan and a backup plan to implement in the future in order to overcome “step”-relevant barriers; D: To identify “step”-relevant strengths; E: To identify “step”-relevant concerns and problems; F: To promote “step”-relevant strengths for the purpose of improving medication adherence; G: To facilitate the couples’ ability to work together and problem-solve regarding “step”-relevant concerns that serve as barriers to adherence; H: To create a participant-driven plan and a backup plan to implement in the future to leverage “step”-relevant strengths for adherence.

**Figure 2:**
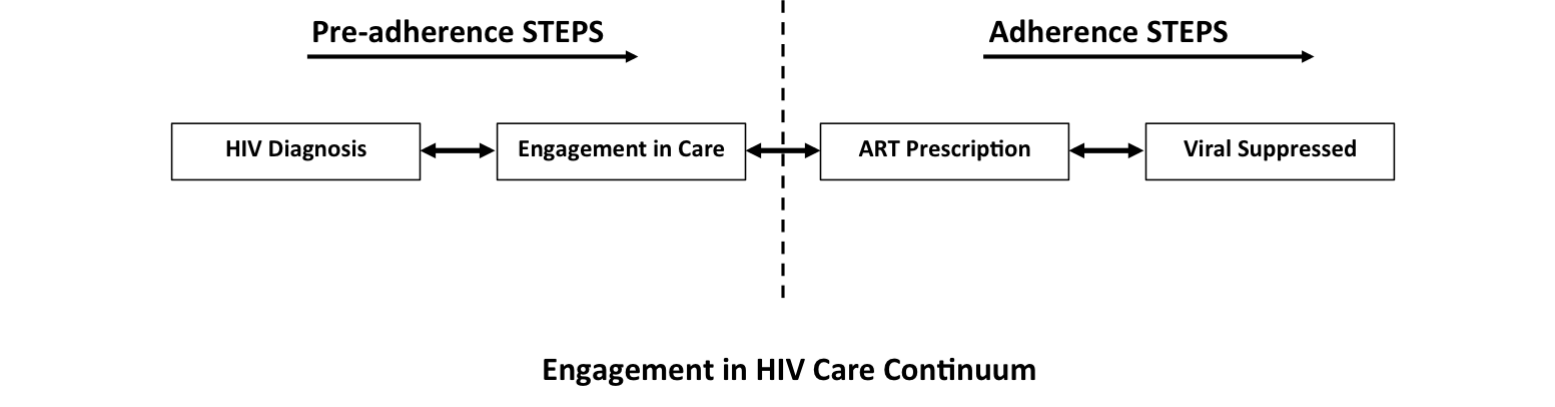
Partner Steps intervention content for the HIV care continuum.

The intervention sessions are structured to cover the following topics: introduction and background, preadherence or adherence steps (specific modules), and review and planning. The manual is also “tracked,” allowing the interventionist to do a quick inventory at baseline (Figure 2; Table 2) to determine where the couple needs to begin. Depending on how far couples have progressed along the HIV care continuum, couples either receive preadherence or adherence steps, and the interventionist can also administer “booster sessions” at 6-month interval follow-up visits to quickly review all material covered in the first session, allowing the couple to receive as many needed “steps” as possible (eg, the dyad needs to cover more than six steps in one session). The manual uses simplified language (ie, layman’s terms) to relate to the broadest audience possible and to avoid distracting or confusing language and unnecessary detail. Lastly, the manual includes worksheets or “takeaways” where participants write down solutions to their adherence blocks and can reference these, facilitating the usage of Partner Steps material outside of the sessions.

**Table 2:** Assessment tool for partner steps preadherence content.

		Yes or No?	
Preadherence steps	Screening and assessment questions	HIV-infected partner	HIV-uninfected partner
1. Coping with HIV	Do either of you feel that you are having trouble or difficulty coping with your HIV diagnosis?	Yes No	Yes No
	Are either of you worried about other people learning about your HIV diagnosis and/or treating you differently?	Yes No	Yes No
2. Health insurance	Are you/your partner having any trouble getting health insurance or are you worried about medical costs?	Yes No	Yes No
3. Health care navigation	Are you unsure who to call or how to navigate the health care system?	Yes No	Yes No
4. Getting to appointments	Is making and remembering appointments difficult?	Yes No	Yes No
5. Transport	Is transportation a challenge?	Yes No	Yes No
6. Housing	Do you not have stable housing right now?	Yes No	Yes No
7. Comfort with providers	Are either of you worried about unfriendly health care providers or staff or how you might be treated?	Yes No	Yes No
8. Provider communication	Are either of you concerned that it might be difficult to talk with health care providers?	Yes No	Yes No
9. Side effects	Are either you concerned about medication side effects?	Yes No	Yes No
10. Lack of interest	Do you feel it’s not necessary to see a doctor or start treatment right now?	Yes No	Yes No
11. Mood management	Do either of you feel that your mood has gotten in the way of seeking HIV care?	Yes No	Yes No
12. Substance use	Has drinking or using drugs gotten in the way of seeking HIV care?	Yes No	Yes No

### Training of Interventionists

Partner Steps interventionists complete a 2-day training comprised of seven training “modules” covering background information, couples counseling skills, intervention content, and opportunities to practice counseling through role-playing scenarios. The first two modules are designed to impart an understanding of the intervention, aims, theoretical framework, and counseling skills. After mastering these concepts and skills, the training modules guide interventionists through all Partner Steps content, including the preadherence and adherence problem-solving “steps.” This phase of the training is discussion-based, interactive, and requires participants to practice newly acquired counseling skills in a series of role-playing scenarios that are observed by trainers. An important emphasis throughout the entire intervention manual and interventionist training protocol is a focus on increasing interaction within dyads, with the ultimate goal of engaging both HIV-negative and HIV-positive partners. Throughout Partner Steps, interventionists work to identify and leverage relationship strengths that can be applied toward helping the couple better problem solve together. The training emphasizes motivational interviewing techniques adapted for a couple’s context, such as both partners completing motivation scales, sharing these with one another, and then discussing their results together to create a unified plan.

### Fidelity Monitoring

In order to monitor fidelity to the Partner Steps manual, we created a two-part system: (1) interventionists complete a checklist immediately following intervention delivery, and (2) all intervention visits are audio-recorded and reviewed by an outside monitor (eg, any intervention-trained study staff member who was not present during the intervention). Both the interventionist checklist and the outside monitoring form contain specific directions to standardize the information reported. The forms asks detailed questions about which required and recommended intervention elements were performed by the interventionist. Required and optional elements that are not performed must be explained and/or justified in the notes section. As the success of the previous Life-Steps intervention hinged on successful problem solving and planning on the part of the interventionist as well as from the participant, the fidelity monitoring plan accounts for these interactions. Thus, for each of the counseling “steps,” the forms require the interventionist to report on a scale with three responses (0 = not sufficiently covered; 1 = somewhat covered; and 2 = sufficiently covered) the level of problem solving and the level of planning that occurred during the visit. The forms also assess the level of engagement from each participant individually and the couple as a whole. This fidelity monitoring plan will enable study staff to improve upon and adapt the intervention based on participant feedback and results from the quality assurance of the outside monitor.

## Discussion

### Summary of Key Innovations

This paper describes the development of Partner Steps, a theory-based intervention to improve engagement in HIV care and ART adherence among HIV serodiscordant and concordant HIV-positive male-male couples. We drew on theory and evidence to develop Partner Steps, which leverages the strengths of men's relationships to address engagement in the entire HIV care continuum, extending beyond previous individual- and couples-based interventions focusing on HIV treatment adherence alone. In order to address the unique needs of male-male couples in which at least one partner is HIV positive, we innovated new content and counseling approaches to better accomplish the aims of this intervention. After describing the content and methods of Partner Steps, which is delivered by trained interventionists to couples through two in-person sessions, several brief phone calls, and booster sessions, we highlight several key innovations and provide recommendations for other researchers interested in using a dyadic approach to HIV prevention.

First, the intervention was designed to be adaptable and applicable for couples at any stage along the HIV care continuum. This flexible design recognizes the necessity of passing through each stage of the continuum in sequence in the current health care system, but also the reality that HIV-positive individuals can regress, cycling through struggles with ART adherence, and falling out of care. As a result, the intervention triages the most important problem solving “steps” according to the couples’ recent HIV care experience. This adaptability is achieved through subdividing the intervention into “preadherence” steps and “adherence” steps and by casting a wide net as to the potential barriers to engaging in HIV care. Although prior couples interventions have focused on one element of the HIV care continuum, this intervention empowers couples to work together in all aspects of HIV care [31,97].

Second, we integrated couples counseling approaches and relationship strength-building exercises to increase the capacity of these couples to work together on problem-solving strategies [32]. The intervention promotes pre-existing and established abilities to accomplish the following: work together as a united team; support each other emotionally and otherwise; cope with hardships; and problem-solve together through challenges. These pre-established skills are then harnessed for the purpose of achieving goals that progress the couple across the HIV care continuum in the direction of the target aim, viral suppression. Though past interventions have worked with couples in similar ways [98], few have tailored the intervention to male-male couples or accounted for a diverse set of aims that relate to the HIV care continuum.

As a third point of innovation, we used a broad definition of “couples” to be inclusive of a more diverse array of male-male couples. Rather than the longer-term inclusion criteria of relationship durations of ≥6 months used in many existing intervention studies, we developed an intervention that would be inclusive of shorter-term couples in which partners felt “committed” to each other “above all others” with relationship duration of at least 1 month. We used this relatively open inclusion criterion, in which couples are not required to be monogamous, legally committed, or in a relationship for a significant amount of time, in order to engage a broad range of couples who could benefit from the intervention. Our study team intentionally limited any assumptions about signifiers of love, trust, intimacy, and relationship strength based upon sexual agreements or relationship duration. We believe that this approach will help maximize accessibility to Partner Steps for many couples who could benefit from the intervention. Prior studies with couples have had narrower eligibility criteria, overlooking partnerships in need of assistance with the HIV care continuum [62].

Finally, Partner Steps is innovative in its accommodation of both HIV serodiscordant and concordant HIV-positive male couples. Two manuals were developed, 1 for each combination of serostatuses. This decision is based upon a large number of considerations related to the elements within the intervention that promote better engagement across the HIV care continuum that concordant HIV-positive couples stand to benefit from as well. Very few interventions in the United States to date have worked with couples in which both partners are HIV-positive and struggling with different aspects of HIV care and treatment [31,97].

**Limitations and Conclusions**

This protocol description is limited by several factors. First, we lack of data on the feasibility, acceptability, and efficacy of the Partner Steps intervention. However, we are currently assessing these factors in 3 sites; thus, the processes and outcomes of the program will be assessed in a diverse population of male couples and by trained staff with slightly different background experiences and qualifications. We acknowledge that future research is needed to identify ideal intervention delivery settings, potential organizational challenges, and interventionists with the capacity to successfully engage and retain couples in the intervention over time. Exploring these aspects of intervention implementation in future research will be critical to the ultimate uptake and success of our intervention. Another limitation is that our preliminary intervention design centered on a target population ≥18 years of age and biologically male; intervention research is urgently needed for younger and transgender populations at risk for HIV acquisition who may benefit from dyad-focused interventions to promote their involvement in the HIV care continuum. Finally, many of the innovations of Partner Steps have the potential to inform a diverse array of ongoing and novel couples-based intervention structures and delivery formats (eg, using online social media) among key populations in diverse geographical settings [98,99]. Despite these limitations, we believe that Partner Steps has high policy relevance, particularly given new recommendations to prescribe ART medications to all adults and adolescents with HIV and a call from the White House Office of National AIDS Policy for innovative strategies to improve progression through the HIV care continuum [50].

## Appendix

Multimedia Appendix 1 Sources for development of Partner Steps adherence content.

Multimedia Appendix 2 Sources for development of Partner Steps preadherence content.

## Abbreviations

ART: antiretroviral therapy
CDC: US Centers for Disease Control and Prevention
CHTC: couples HIV testing and counseling
HIV: human immunodeficiency virus
MSM: men who have sex with men
PrEP: pre-exposure prophylaxis
