# Supplementary material for: A Dyadic Behavioral Intervention to Optimize Same Sex Male Couples’ Engagement Across the HIV Care Continuum: Development of and Protocol for an Innovative Couples-based Approach (Partner Steps)
Source: JMIR Res Protoc. 2016 Aug 25;5(3):e168. doi: 10.2196/resprot.6271 (PMC5016626; doi:10.2196/resprot.6271)
Supplement: Multimedia Appendix 2 [file resprot_v5i3e168_app2.pdf]

**Multimedia Appendix 1:** Sources for Development of Partner Steps Pre-Adherence Content

| **Pre-Adherence Steps:** | **1. Coping with HIV** | **2. Health insurance** | **3. Healthcare navigation** | **4. Trouble remembering to make or attend appointments** | **5. Transportation** | **6. Trouble with housing** | **7. Comfort with providers** | **8. Communicating with providers** | **9. Fear of drug side effects** | **10. Lack of interest in receiving care** | **11. Managing mood** | **12. Substance use** |
|---|---|---|---|---|---|---|---|---|---|---|---|---|
| IAPAC Guidelines | X | X | X | X | X | X | X | X | X | X | X | X |
| White House Care Continuum Initiative | X | X | X | X | X | X | X | X | X | X | X | X |
| Literature Review | X | X | X | X | X | X | X | X | X | X | X | X |
| CHTC Skills | X | | | | | | | | | | | |
| Life-Steps: getting to appointments | | | | X | X | | | | | | | |
| Life-Steps: communication with physicians and other medical personnel | | | | | | | X | X | | | | |
| Life-Steps: coping with side effects | | | | | | | | | X | | | |
