# Supplementary material for: A Dyadic Behavioral Intervention to Optimize Same Sex Male Couples’ Engagement Across the HIV Care Continuum: Development of and Protocol for an Innovative Couples-based Approach (Partner Steps)
Source: JMIR Res Protoc. 2016 Aug 25;5(3):e168. doi: 10.2196/resprot.6271 (PMC5016626; doi:10.2196/resprot.6271)
Supplement: Multimedia Appendix 1 [file resprot_v5i3e168_app1.pdf]

Multimedia Appendix 2: Sources for Development of Partner Steps Adherence Content

Adherence Steps												
	**Introduction**	**1. Transportation to appointments**	**2. Obtaining medications**	**3. Communicating with providers**	**4. Storing & Transporting Medications**	**5. Coping with side effects**	**6. Having a daily medication schedule**	**7. Adherence, self-care & your relationship**	**8. Communicating within your relationship**	**9. Managing your social life & other relationships**	**10. Dealing with privacy & disclosure**	**Review & Planning**
IAPAC Guidelines		X	X	X	X	X	X					
White House Care Continuum Initiative	X											
Literature Review	X	X	X	X	X	X	X	X	X	X	X	
CHTC Skills	X							X	X	X	X	
Life-Steps: psychoeducation												X
Life-Steps: getting to appointments		X										
Life-Steps: communication with physicians and other medical personnel				X								
Life-Steps: coping with side effects						X						
Life-Steps: obtaining medications			X									
Life-Steps: concrete daily medication schedule							X					
Life-Steps: storing of medications					X							
Life-Steps: cue-control strategies							X					
Life-Steps: handling slips												X
Life-Steps: review												
